# Production of a Bioengineered G-Protein Coupled Receptor of Human Formyl Peptide Receptor 3

**DOI:** 10.1371/journal.pone.0023076

**Published:** 2011-08-11

**Authors:** Xiaoqiang Wang, Shuguang Zhang

**Affiliations:** 1 Laboratory for Molecular Fabrication, Center for Bits and Atoms, Massachusetts Institute of Technology, Cambridge, Massachusetts, United States of America; 2 Center for Bioengineering and Biotechnology, China University of Petroleum (East China), Qingdao, Shandong, China; Universität Heidelberg, Germany

## Abstract

G-protein coupled receptors (GPCRs) participate in a wide range of vital regulations of our physiological actions. They are also of pharmaceutical importance and have become many therapeutic targets for a number of disorders and diseases. Purified GPCR-based approaches including structural study and novel biophysical and biochemical function analyses are increasingly being used in GPCR-directed drug discovery. Before these approaches become routine, however, several hurdles need to be overcome; they include overexpression, solubilization, and purification of large quantities of functional and stable receptors on a regular basis. Here we report milligram production of a human formyl peptide receptor 3 (FPR3). FPR3 comprises a functionally distinct GPCR subfamily that is involved in leukocyte chemotaxis and activation. The bioengineered FPR3 was overexpressed in stable tetracycline-inducible mammalian cell lines (HEK293S). After a systematic detergent screening, fos-choline-14 (FC-14) was selected for subsequent solubilization and purification processes. A two-step purification method, immunoaffinity using anti-rho-tag monoclonal antibody 1D4 and gel filtration, was used to purify the receptors to near homogeneity. Immunofluorescence analysis showed that expressed FPR3 was predominantly displayed on cellular membrane. Secondary structural analysis using circular dichroism showed that the purified FPR3 receptor was correctly folded with >50% α-helix, which is similar to other known GPCR secondary structures. Our method can readily produce milligram quantities of human FPR3, which would facilitate in developing human FPR as therapeutic drug targets.

## Introduction

G-protein coupled receptors (GPCRs), also referred to as seven-transmembrane (7TM) domain receptors, although functionally diverse, constitute the largest integral membrane protein family in the human genome [1]–[3]. Members of GPCR family share a common topological structure on cellular membrane that contains 7 transmembrane helices with an extracellular N-terminus and an intracellular C-terminus connected by three intracellular loops and three extracellular loops [1], [2]. Based on sequence homology, ligand structure or receptor function, GPCRs are classified into more than one hundred subfamilies. These receptors are physiologically important in humans, participating in the regulation of most of our physiological actions such as neurotransmission, enzyme release, chemotaxis or inflammation, as well as our sense of vision, smell and taste, by sensing endogenous or environmental stimuli through binding compatible ligands and transducing corresponding signal into cells typically through coupled heterotrimeric G protein. Hence GPCRs are the most frequently addressed therapeutic targets for a number of disorders and diseases. Currently ∼50% of all prescription drugs and ∼66% of drugs in development are targets for GPCRs [3]–[5].

Formyl peptide receptors (FPRs) comprise a functionally distinct GPCR subfamily involved in leukocyte chemotaxis and activation. In human and other primates, 3 FPR subtypes have been identified [6]. The first defined human FPR gene, FPR1 (www.uniprot.org/uniprot/P21462), was initially reported as a high affinity binding site on the surface of neutrophils for the prototypic N-formyl peptide formyl-methionine-leucine-phenylalanine (fMLF) and then its gene was cloned in 1990 from a differentiated HL-60 myeloid leukemia-cell cDNA library [7], [8]. Two additional human FPRs, designated FPR2 (www.uniprot.org/uniprot/P25090) and FPR3 (www.uniprot.org/uniprot/P25089), were subsequently cloned by low-stringency hybridization using the FPR1 cDNA as a probe. These FPR genes are shown to cluster on human chromosomal region 19q13.3 [9]–[11].

Functionally, these receptors are considered to play important roles in innate immunity and host defense mechanisms, including immune response to microorganism infection, proinflammatory response in amyloidogenic diseases, host responses to cell necrosis and apoptosis and so on [6], [12], [13]. These receptors may also influence the expression and function of CCR5 and CXCR4 on human monocytes, two major GPCR chemokine coreceptors of human immunodeficiency virus type 1 (HIV-1) [14], [15]. Moreover, human FPR expression has been observed in numerous distinct tissues and cell types ([6]–[7] and references therein), indicating a much broad distribution of these receptors and their physiologically significant role *in vivo*. Hence FPRs comprise an appealing group of therapeutic targets, most importantly, for infectious and immunologically mediated diseases.

We selected FPR3 for our study because a peptide ligand is known for FPR3. Also FPR3 shares 56% and 83% amino acid sequence identity to FPR1 and FPR2, respectively. Interestingly, the FPR3 does not respond to fMLF, the prototypic N-formyl peptide usually generated at sites of bacterial infection or tissue injury, while FPR1 binds fMLF with high affinity and FPR2 does with low affinity [16], [17]. This phenomenon indicates that the N-formyl group is not essential for ligand binding to human FPRs. In addition, compared to FPR1 and FPR2, a small number of ligands for FPR3 have been identified, among which is an endogenous acetylated peptide F2L. This F2L appears to be the most specific ligand that binds and activates FPR3 in the low nanomolar range [18]. Ongoing efforts are still required to further expand the ligand spectrum of FPR3, to decipher the underlying structural and functional mechanisms of FPR3 as well as two other human FPRs, and to eventually identify novel pharmacologically relevant interacting partners.

The classical techniques employed in GPCR-directed drug discovery are chiefly cell-based assays combined with high throughput screening (HTS) method. These techniques are suited to measure GPCR ligand binding, G-protein function and second messenger generation, and GPCR downstream signaling [19]. As an alternative to the cell-based assays, purified GPCR-based approach becomes increasingly attractive in GPCR-directed drug discovery. By using stabilized pure GPCRs, diffraction-quality protein crystals can be obtained, which would lead to an insight into GPCR structure that may eventually identify each unique functional property. Although this is a challenging goal, 6 molecular structures of GPCRs have been resolved, including bovine rhodopsin [20], [21], human β_2_-adrenergic receptor [22], [23], avian β_1_-adrenergic receptor [24] and human A_2A_ adenosine receptor [25], human CXCR4 [26] and human dopamine D3 receptor [27]. The availability of additional GPCR structures, along with novel biophysical and biochemical approaches to directly study purified GPCRs, would help gain a deep understanding of GPCRs' structure and function relationship, and also further stimulate drug screening and structure-based drug design [28].

In order to carry out study of FPR3 as well as purified protein-based biophysical and biochemical function analyses, large quantities of active and stable receptors are required. Because of the extremely low abundance of most GPCRs in their natural environments, overexpression in heterologous host systems is a prerequisite for large quantity production.

Here we report the expression and purification of a bioengineered human FPR3 from stable tetracycline-inducible HEK293S cell lines, a heterologous expression system suitable for large-scale GPCR protein productions [29]–[31]. After a systematic detergent screening, fos-choline-14 (FC-14) was selected for subsequent solubilization and purification processes. Our previous study identified FC14 as one of the best detergents [30]–[32]. A two-step purification method using monoclonal antibody immunoaffinity purification and gel filtration was employed to purify the receptors to near homogeneity. Immunofluorescence analysis indicated that expressed FPR3 was predominantly distributed on cellular membrane. Furthermore secondary structural analysis using circular dichroism (CD) showed that the purified FPR3 receptors were correctly folded with >50% α-helical content, which is consistent with other known GPCR α-helical content. Using this method, we obtained milligram quantities of human FPR3, which would be used for further studies.

## Results

### Construction of stable human FPR3-inducible HEK293S cell lines

**Figure 1:**
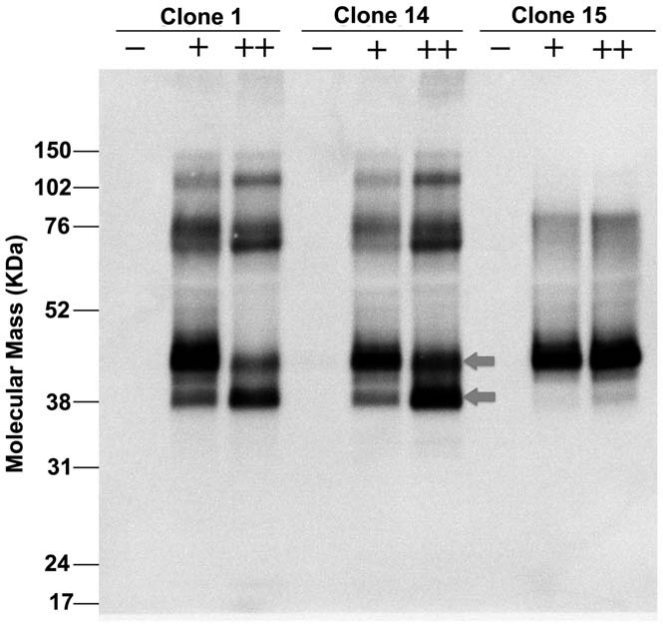
Construction of stable human FPR3-inducible HEK293S cell lines using the T-REx system (Life Technologies). Clones were tested for induction after 48 h in plain media (−) or media supplemented with 1 µg/ml tetracycline (+) or tetracycline plus 2.5 mM sodium butyrate enhancer (++). Arrows indicate the positions of the 38- and 42-kDa monomer forms. Levels of human FPR3 were probed via Western blot analysis against the rho1D4 tag at the C-terminus of the protein sequence. Clones 1, 14 and 15 show highest levels of induction while maintaining undetectable background levels in the absence of tetracycline. Clone 15 that only expressed the 42-kDa FPR3 monomer was selected for subsequent experiments.

The stable human FPR3-inducible HEK293S cell lines [29]–[31] were constructed using the Invitrogen T-REx tetracycline regulation system as described before [29]–[30]. In all, 30 clones were screened for FPR3 expression for selecting stable cell lines. Figure 1 shows the Western blot analysis result of FPR3 expression in clone 1, clone 14 and clone 15, respectively, which among all the clones showed the highest expressions after 48-hour induction in media supplemented with 1 µg/ml tetracycline. It can be seen that in these 3 clones, no apparent background levels are detected by Western blot analysis using anti-rho-tag monoclonal antibody 1D4 in the absence of tetracycline induction. Whereas, FPR3 expression of clone 14 and clone 15 was more enhanced after induction using tetracycline combining with sodium butyrate than using tetracycline alone. The positive synergistic effect of sodium butyrate on expression, when used with tetracycline regulation system, has been demonstrated previously in the study of several other GPCRs [29]–[31].

Western blot analysis using a monoclonal antibody against the C-terminal rho-tag 1D4 tag also revealed two immunoreactive monomeric FPR3 bands in both clone 1 and clone 14, which migrated at approximately 38 kDa and 42 kDa, respectively, indicated by arrows. Although the bioengineered FPR3 has a theoretical molecular weight of 42.4 kDa, however, it is common that two monomeric FPR3 forms migrate slightly faster than expected on SDS-PAGE gel, like many other membrane proteins [29]–[31]. For both clone 1 and clone 14, while the 42 kDa band predominated after 48-hour induction in media supplemented with 1 µg/ml tetracycline, the 38 kDa band predominated over the 42 kDa band after 48-hour induction in media supplemented with 1 µg/ml tetracycline plus 2.5 mM sodium butyrate. Similar phenomena have been observed in our previous study of another GPCR of human olfactory receptor 17-4 [30]–[31].

By contrast, for clone 15, the 42 kDa is the only visible band under both induction conditions. Clone 15 also produced much less dimeric and oligomerized FPR3 receptors than clone 1 and clone 14, as revealed on the upper part of corresponding lanes. Because protein heterogeneity could potentially interfere with future crystallization and structural study, we thus only selected clone 15 for all subsequent experiments. The concentrations of tetracycline and sodium butyrate were also optimized with respect to FPR3 expression level for clone 15. A combination of 1 µg/ml tetracycline and 2.5 mM sodium butyrate was optimal for FPR3 expression, and thus this induction condition was selected for all subsequent expressions.

### Detergent screening of human FPR3 solubilization from HEK293S cells

The selection of a suitable detergent for membrane protein solubilization from cellular membrane and its stabilization is crucial not only for subsequent purification process, but also for membrane protein activity preservation and the final protein yield. However, our understanding of the interaction between detergents and membrane proteins is still limited, and currently the choice of a detergent is mostly empirical. Indeed an elaborate and laborious screening process is required to select an optimal detergent, which usually results in membrane protein-specific optimization.

Although fos-choline series were found most effective for solubilizing and stabilizing several other GPCRs after systematic detergent screening in our lab [30]–[32], it still remained unknown if these detergents would work well for human FPR3 receptors. Thus, we again carried out a systematic detergent screening that included representatives from non-ionic, anionic, cationic and zwitter-ionic detergent classes, and some detergent mixtures that are proven effective for solubilizing other GPCRs.

**Figure 2:**
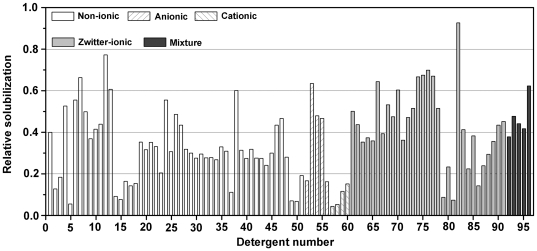
Detergent screening for optimal solubilization of human FPR3 expressed in HEK293S cells. Expression of the FPR3 was induced with tetracycline (1 µg/ml) and sodium butyrate (2.5 mM) for 48 h and receptors were solubilized in PBS containing detergent(s) for 1 h at 4°C. In total, 96 detergents/detergent mixtures were screened in this study. Each integer from 1 to 96 along x-axis represents a detergent/detergent mixture (see Table S1 for more information about each detergent/detergent mixture). The ability of screened detergents/detergent mixtures to solubilize the FPR3 has been quantified by spot densitometry and normalized to the range from 0.0 to 1.0 for ease of comparison.

The detergent screening result is shown in Figure 2, with 96 detergents/detergent mixtures screened and their solubilization ability quantified by spot densitometry. The 3 best detergents found most effective for solubilizing the FPR3 included Fos-choline-unsat-11-10 (zwitter-ionic), Anapoe-X-100 (non-ionic) and Fos-choline-14 (zwitter-ionic). Again, Fos-choline-unsat-11-10 and Fos-choline-14 (FC-14) from fos-choline series were shown to be very effective for solubilizing the FPR3 receptors from HEK293S cells, which are structurally related to phosphatidylcholine (PC), a phospholipid and major constituent of the lipid bilayer of mammalian cells.

In order to act as an effective solubilizer for membrane protein study, a detergent must be used at concentrations above its critical micelle concentration (CMC), namely, the minimal concentration required for detergent monomers to self-assemble into non-covalent micelles. In general, the CMC dictates how much detergent is needed in the solubilization and stabilization of membrane protein. Fos-choline-unsat-11-10 has a high CMC value of 0.21%, although it is found to be the most effective detergent among the 96-detergents/detergent mixtures screened in this study. In contrast, Anapoe-X-100 and FC-14 also very effective for solubilizing the FPR3 have much lower CMC values of 0.015% and 0.0046%, respectively. For consideration of cost-effectiveness in detergent usage, FC-14 was selected for all subsequent solubilization and purification, which was also used in our previous studies of other GPCRs and shown also very effective for stabilizing GPCRs [30]–[32].

### Purification of human FPR3 produced from HEK293S cells

The purification of solubilized receptors is another major obstacle to overcome in large-quantity GPCR protein production. It is usually very difficult to achieve efficient purification if using conventional ion-exchange chromatography. In this study, we used a two-step purification method comprising first immunoaffinity purification and then gel filtration to purify the FC-14 solubilized FPR3 receptors, which was shown to be highly efficient in the purification of other GPCRs [30]–[32]. For the first immunoaffinity purification step, CNBr-activated Sepharose 4B beads linked to the mouse monoclonal rho1D4 antibody were used to capture FC-14 solubilized FPR3. Following a thorough wash procedure to remove non-specific impurities, the captured receptors were eluted by the addition of an excess of rho1D4 elution peptide (TETSQVAPA). Western blot analysis indicated that the receptors eluted primarily in the first 3-elution fractions after being captured completely by the rho1D4 antibody bead matrix.

**Figure 3:**
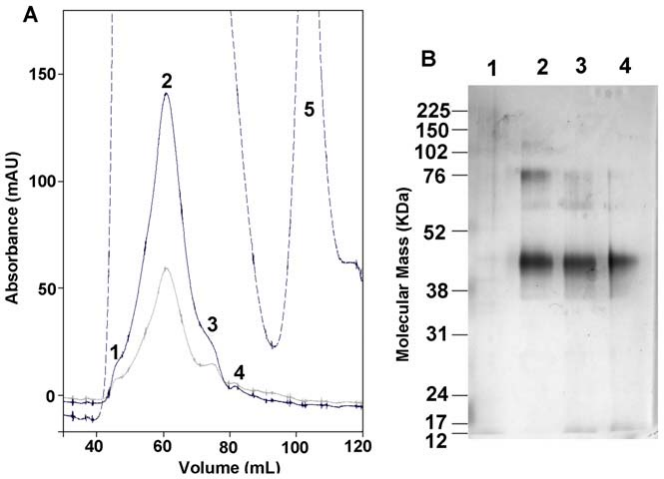
A) Gel filtration on immunoaffinity-purified human FPR3 from ∼6 g cells (wet weight). Absorbance was recorded at 280 nm (black line), 254 nm (gray line), and 215 nm (dashed line). Peak 1–4 (indicated by numbers) were pooled and concentrated. Peak 5 contained the rho1D4 elution peptide TETSQVAPA from the immunoaffinity purification. B) Silver staining of the peak fractions. Peak numbers refer to those designated in (A).

To further purify the receptor and to remove the elution rho1D4 peptide, the elution fractions from immunoaffinity purification were concentrated and then subjected to gel filtration on a Äkta HPLC system. Column flow-through was monitored by UV absorption (280 nm, 254 nm and 215 nm) and separated into fractions by an auto-fraction collector. Shown in Figure 3A is the chromatogram of gel filtration on immunoaffinity-purified human FPR3 from ∼6 g induced HEK293S cells [29]–[31], in which several distinct peaks can be observed. The peak fractions were then pooled, concentrated and subjected to SDS-PAGE followed by Western blot and total protein staining. Figure 3B shows the result of protein staining analysis on the peak fractions. Peak 2 contained predominantly monomeric FPR3 (>90% purity) and also a very small amount of dimeric FPR3. Earlier peak 1 contained no samples that could be detectable by total protein staining. The later peaks 3 and 4 mainly contained monomeric FPR3 and a truncated form of FPR3 (∼15 kD). Peak 5 contained the rho1D4 elution peptide from the immunoaffinity purification. The final yield of purified FPR3 monomer was ∼2 mg. Thus, using a simple two-step method, we have successfully obtained 2 milligrams of human FPR3.

### Cellular localization of human FPR3 using Immunofluorescence

**Figure 4:**
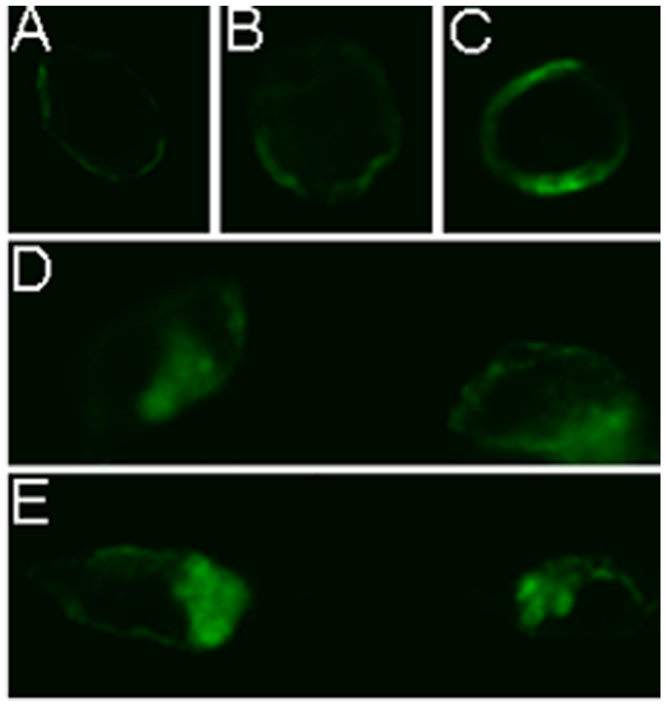
Immunofluorescence analysis of cell surface expressed human FPR3. HEK293S cells were induced for 48 h in media supplemented with 1 µg/ml tetracycline plus 2.5 mM sodium butyrate. Cell images were taken after being fixed and incubated with monoclonal anti-rho1D4 antibody followed by Alexa Fluor 488 goat anti-mouse antibody.

Cell-based immunofluorescence analysis was also performed to verify the distribution of expressed FPR3 on cellular membrane. First, HEK293S cells [29]–[31] were induced for 48-hour in media supplemented with 1 µg/ml tetracycline plus 2.5 mM sodium butyrate. The cells were then fixed and permeabilized, followed by blocking with bovine serum albumin. Mouse monoclonal rho1D4 antibody was then applied to HEK293S cells, followed by Alexa Fluor 488 goat anti-mouse antibody. Cells were then examined using excitation at 488 nm. Figure 4 shows the immunofluorescence images of induced HEK293S cells. Enhanced fluorescence can be seen around the edge of the cells, indicating the expressed FPR3 was predominantly distributed on plasma membrane.

### Secondary structural studies using circular dichrism of purified human FPR3

**Figure 5:**
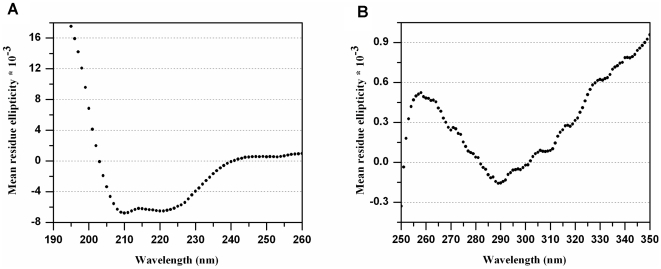
Secondary structural study of the purified human FPR3 monomer using both far-UV and near-UV CD spectroscopy. Mean residue ellipticity [θ] has units of degree×cm^2^×dmol^−1^. A) Far-UV CD spectrum of FPR3 displaying secondary structure of ∼57% α-helix. B) Near-UV CD spectrum of FPR3 showing distinct tertiary structure profiles. Spectra shown are the average of 3 replicate scans.

We next asked whether the purified, FC-14-solubilized human FPR3 retained properly folded secondary structure. The α-helical feature of GPCRs has been confirmed by the known molecular structures with high resolution [20]–[27]. The computer prediction for wild-type human FPR3 secondary structure, based on a free web based transmembrane domain calculations, www.uniprot.org/uniprot/P25089, is ∼43% α-helix. 0 When we measured the FPR3 using far-UV CD spectroscopy, the monomeric FPR3 displayed a spectrum characteristic of a predominantly characteristic α-helical protein with minima at 208 nm and 222 nm (Figure 5A). Analysis of the spectrum using the K2D2 algorithm (www.ogic.ca/projects/k2d2/) showed that FPR3 has ∼57% α-helical content. This α-helical content is higher than the computer predicted value. This higher value is likely from the contribution of other α-helical segments in addition to the standard 7-transmembrane domains. Likewise, several distinct profiles were observed in near-UV CD spectroscopy (Figure 5B), which suggests a defined tertiary structure for the FPR3. It is known that the functional bovine rhodopsin also has similar near-UV profile in this region; whereas nonfunctional opsin mutants show flat spectra characteristic of a misfolded globular state [33]. Thus both far-UV and near-UV CD spectra suggest that the bioengineered human FPR3 folded correctly after purification and is suitable for subsequent structural and functional studies.

## Discussion

Formyl peptide receptors (FPRs) comprise a functionally distinct GPCR subfamily involved in leukocyte chemotaxis and activation. The broad tissue distribution of human FPRs and their potential physiological roles make them an appealing group of therapeutic targets, most importantly, for infectious and immunologically mediated diseases.

Currently, as an alternative to the cell-based assays, purified GPCR-based approach is increasingly being used in GPCR-directed drug discovery. To initiate structural study and biophysical and biochemical analyses directly exploiting purified proteins for FPR-focused drug discovery, large-quantity production is often required.

In this study, we successfully constructed human FPR3-inducible mammalian cell lines and produced milligram quantities of high quality FPR3 receptors following the method utilized for large-scale production of hOR17-4 [30]–[31]. To our knowledge, this is the first time a human FPR has been heterologously expressed and purified in millilgrams.

To achieve receptor overexpression but minimize the toxic effects, the stable human FPR3-inducible HEK293S cell lines [29]–[31] were first constructed using the Invitrogen T-REx tetracycline regulation system as described before [29]–[31]. This allowed the HEK293S cells [29]–[31] to be grown to the desired density, when needed, production of freshly produced receptor can be induced by the addition of tetracycline. Thus structurally and functionally homogenous FPR3 receptors could be obtained. Without tight regulation system, the receptor production may be compromised due to constitutive expression in HEK293S cells [29]–[31]. To achieve receptor overexpression, altogether 30 clones were screened and compared, among which clone 1, clone 14 and clone 15 gave the highest expressions under the same induction condition. For all clones screened, there is no background expression detectable by Western blot analysis in the absence of tetracycline, thus verifying the tightness of the tetracycline regulation system.

It is interesting to notice that the FPR3 monomer expressed in clone 1 and clone 14 migrated as a doublet at approximately 38 kDa and 42 kDa. Both monomer forms of the expressed FPR3 receptor are believed to be intact, full-length proteins, since both of them were detected by the C-terminal rho1D4 monoclonal antibody. In our previous study on hOR17-4 production, two monomeric hOR17-4 bands were also detected, which was demonstrated to be caused by glycosylation as this size discrepancy was eliminated by mutation of the glycosylation site [30]–[31]. The size discrepany of expressed FPR3 monomers is likely also caused by glycosylation and the 42 kDa band possibly constituted a glycosylated form of the receptor. Actually, human FPR3 is predicted to have 3 N-glycosylation sites [34] and it is possible that glycosylation occurred at more than one potential sites in the 42 kDa FPR3 monomer, which as a result shows a 4 kDa size difference from the 38 kDa monomer. However, further studies are required to fully elucidate the size discrepany of expressed FPR3 monomers.

Moreover, for both clone 1 and clone 14, while the 42 kDa band predominated after induction with tetracycline alone, the 38 kDa band predominated over the 42 kDa band after induction with tetracycline plus sodium butyrate. One explanation is that in clone 1 and clone 14, most receptors are glycosylated after induction with tetracycline alone but the use of sodium butyrate in conjunction with tetracycline, which usually leads to more rapid and higher expression, results in the accumulation of FPR3 receptors in the endoplasmic reticulum (ER) and improper processing of the receptors. Similar phenomenon has been observed previously in the induction of hOR17-4 [30]–[31]. Nevertheless, we observed that such expression mode is cell line-dependent for FPR3 induction. For example, clone 15 also giving high expression level only expressed 42 kDa monomer under both induction conditions tested. Since protein heterogeneity could pose a problem for future crystallization and structural study, we thus choose clone 15 for all subsequent experiments. As loss of glycosylation has bee demonstrated to affect the function of rhodopsin [35], the selection of clone 15 to exclusively express 42 kDa FPR3 monomer that is potentially glycosylated may better suit functional study as well.

Following construction of stable cell lines overexpressing FPR3 receptors suitable for structural and functional studies, the selection of an appropriate detergent is also crucial to subsequent purification, protein activity preservation and the final protein yield. After a systematic detergent screening, several detergents were found most effective for solubilizing the FPR3, including Fos-choline-unsat-11-10 and FC-14 from fos-choline series. However, FC-14 was eventually selected for FPR3 solubilization from HEK293S cells [29]–[31] and its subsequent purification out of consideration for both solubilization- and cost-effectiveness in detergent usage. The latter consideration is based on detergent CMC, the minimal concentration above which a detergent must be used to act as an effective solubilizer in membrane protein study and which as a result also dictates predominantly how much detergent is needed. So far, foscholine series were found very effective for solubilizing and stabilizing all the GPCRs studied in our lab [30]–[32] and by others [36], which are structurally related to phosphatidylcholine (PC), a phospholipid and major constituent of the lipid bilayer of mammalian cells. Thus FC14 may find much broader use in GPCR study in the long run.

After achieving human FPR3 overexpression in stable HEK293S cell line [29]–[31] and then selection of the suitable detergent, we also successfully purified FPR3 to high homogeneity by employing a two-step purification method [29]–[31]. We have already obtained about ∼2 mg FPR3 monomers from ∼6 g induced HEK293S cells [29]–[31]. Secondary structural analysis showed that the purified FPR3 was correctly folded into characteristic α-helix, similar as other known GPCRs. Crystallization trials have already been initiated on human FPR3. Meanwhile, the constructed inducible cell lines are being grown on a large-scale to obtain enough receptors for subsequent studies.

Our approach to produce a high yield of purified human FPR3 is a step forward toward obtaining a large quantity of this receptor not only for structural and functional studies but also for developing human FPR as therapeutic drug targets in the future.

## Materials and Methods

### Gene Construction

The 353 amino acid sequence of human FPR3 was obtained from NCBI online database (accession number NP_002021.3). To adapt the synthetic FPR3 gene for use in mammalian cell expression, protein detection and purification, the following sequence modifications were made: 1) human codon optimization; 2) addition of a C-terminal rho1D4 epitope tag (TETSQVAPA) preceded by a GG linker to facilitate detection and purification; 3) addition of a Kozak consensus sequence (GCCACCACC) immediately 5′ to the ATG start codon; 4) addition of an EcoRI restriction site at the 5′ end and an XhoI restriction site at the 3′ end of the gene to enable cloning into expression vectors. The FPR3 gene was then commercially synthesized and subcloned into the T-REx pcDNA4/To inducible expression plasmid (Invitrogen, Carlsbad, CA) by GENEART. The final construct was verified by DNA sequencing and used for all subsequent studies.

### Construction of Stable Inducible Cell Lines

The stable human FPR3-inducible HEK293S cell lines were created as described before [29]–[31]. Briefly, HEK293S cells containing the stable expression of pcDNA6/Tr (Invitrogen) were grown in regular medium to form monolayers at 37°C at 5% CO_2_. The pcDNA4/To plasmid containing the optimized human FPR3 gene was then transfected into these cells using Lipofectamine 2000. After 48 hours cells were subjected to drug selection in 5 µg/ml blasticidin and 50 µg/ml zeocin for 2–3 weeks, and then subcloned with the zeocin concentration reduced to 25 µg/ml from now on. 30 clones were expanded and screened for inducible expression of human FPR3. For each clone, three parallel expressions were tested: after 48 h in plain media (−) or media supplemented with 1 µg/mL tetracycline (+) or tetracycline plus 2.5 mM sodium butyrate enhancer (++). Expression was assayed via Dot blot and Western blot using the mouse monoclonal antibody rho1D4. Clones showing the highest-level expression of FPR3 under induction conditions while maintaining undetectable expression without induction were selected and expanded into large-scale culture and used for all subsequent experiments.

### Systematic Detergent Screening

In total, 96 detergents/detergent mixtures were screened here, with 88 detergents chosen from the Solution Master Detergent Kit (Anatrace). Additionally, HEGA-10, NP-40, Digitonin and five detergent mixtures were also chosen because of their effectiveness in the solubilization of other GPCRs. More information about each detergent/detergent mixture was provided in Table S1. The detergent screening process has been described before [31].

### Western Blot, Dot Blot and Total Protein Staining Analyses

For Western blot, samples were first prepared and loaded in Novex 10% Bis-Tris SDS-PAGE gel (Life Technologies) according to manufacturer's protocol, with the exception that the samples were incubated at room temperature prior to loading as boiling caused membrane protein aggregation. The Full Range Rainbow (GE Healthcare, Waukesha, WI) molecular weight marker was loaded as the protein size standard. After samples were resolved on SDS-PAGE gel, they were transferred to a 0.45 µm nitrocellulose membrane, blocked in milk (5% non-fat dried milk in TBST) for 1 hour, and incubated with a rho1D4 primary antibody (1∶3000, 1 hour, room temperature). The target proteins were then detected with a goat anti-mouse HRP-conjugated secondary antibody (Pierce, Rockford, IL) (1∶5000, 1 hour, room temperature) and visualized using the ECL-Plus Kit (GE Healthcare). For Dot blot, 1 µl of each sample was directly pipetted onto 0.45 µm nitrocellulose membrane. After air drying for 20 minutes, the same procedures were performed for target protein detection and visualization as in Western blot analysis. For total protein staining, SDS-PAGE gels were run as described above and then stained using silver staining (a much more sensitive alternative to Coomassie; Invitrogen). All Western blot, Dot blot and total protein staining images were captured using a Fluor Chem gel documentation system (Alpha Innotech, San Leandro, CA).

### Immunoaffinity Purification and Gel Filtration Purification

For immunoaffinity purification, we utilized rho1D4 monoclonal antibody (Cell Essentials, Boston, MA) chemically linked to CNBr-activated Sepharose 4B beads (GE Healthcare). The rho1D4 elution peptide Ac-TETSQVAPA-CONH2 was synthesized by CPC Scientific Inc., CA. Rho1D4-Sepharose immunoaffinity purification has been described [30]. Gel filtration chromatography was employed for further purification using a HiLoad 16/60 Superdex 200 column on a Äkta Purifier FPLC system (GE Healthcare), as described before [30]–[31].

### Immunofluorescence analysis

Cells were plated at a low density in coverslip-bottomed 6-well tissue culture plate. Mouse monoclonal rho1D4 antibody was diluted 1∶1200. Alexafluor-488 goat anti-mouse antibody (Invitrogen) was used at a 1∶3000 dilution. Cells induced for 48 h in media supplemented with 1 µg/ml tetracycline plus 2.5 mM sodium butyrate were first fixed in 10% neutral buffered formalin solution (Sigma-Aldrich) for 20 minutes at room temperature and then permeabilized in ice-cold 1∶1 acetone∶ methanol mixture at −20°C for 3 minutes. Cells were then blocked in PBS with 0.3 M glycine and 4% bovine serum albumin for 1 hour and washed with PBS, followed by overnight incubation at 4°C in PBS with 2% bovine serum albumin and mouse monoclonal rho1D4 antibody. After three washes with PBS, cells were then incubated with Alexafluor-488 goat anti-mouse antibody for 1 hour and washed with PBS. Cells were examined with a Nikon Eclipse TE300 inverted microscope using excitation at 488 nm.

### Secondary structural analysis using circular dichroism

Spectra were recorded at 15°C with a CD spectrometer (Aviv Associates model 410). Far-UV CD spectra were measured over the wavelength range of 195 nm to 260 nm with a step size of 1 nm and an averaging time of 5 seconds. Near-UV CD spectra were measured over the wavelength range of 250 nm to 350 nm. Spectra for purified human FPR3 monomers were blanked to wash buffer. Spectra were collected with a 111-QS quartz sample cell (Hellma) with a path length of 1 mm. The secondary structural content was estimated by using the program K2D2 (www.ogic.ca/projects/k2d2/). Raw data were first smoothed by using the built-in smoothing function of Aviv software and the CD spectra shown here were drawn from the smoothed data. A manual smoothing was performed using 5-degree sliding polynomial.

## Supporting Information

Table S1 **A list of the detergents screened for optimal solubilization of human FPR3 expressed in HEK293 cells.**
(DOC)
