# Supplementary material for: Production of a Bioengineered G-Protein Coupled Receptor of Human Formyl Peptide Receptor 3
Source: PLoS One. 2011 Aug 11;6(8):e23076. doi: 10.1371/journal.pone.0023076 (PMC3154916; doi:10.1371/journal.pone.0023076)
Supplement: Table S1 — A list of the detergents screened for optimal solubilization of human FPR3 expressed in HEK293 cells. (DOC) [file pone.0023076.s001.doc]

Supporting Information

**Table S-1 A list of the detergents screened for optimal solubilization of human FPR3 expressed in** HEK293 cells.

| # | Detergent | Type^1^ | CMC (%)^2^ | Conc.^3^ | Spot intensity^4^ |
| --- | --- | --- | --- | --- | --- |
| 1 | ANAMEG® -7 | N | 0.65 | 2% | 3.30E+06 |
| 2 | ANAPOE®-20 | N | 0.0072 | 2% | 1.05E+06 |
| 3 | ANAPOE®-35 | N | 0.001 | 2% | 1.52E+06 |
| 4 | ANAPOE®-58 | N | 0.00045 | 2% | 4.35E+06 |
| 5 | ANAPOE®-80 | N | 0.0016 | 2% | 453964 |
| 6 | ANAPOE®-C10E6 | N | 0.025 | 2% | 4.58E+06 |
| 7 | ANAPOE®-C10E9 | N | 0.053 | 2% | 5.48E+06 |
| 8 | ANAPOE®-C12E8 | N | 0.0048 | 2% | 4.12 E+06 |
| 9 | ANAPOE®-C12E9 | N | 0.003 | 2% | 3.05 E+06 |
| 10 | ANAPOE®-C12E10 | N | 0.2 | 2% | 3.43 E+06 |
| 11 | ANAPOE®-C13E8 | N | 0.0055 | 2% | 3.63 E+06 |
| 12 | ANAPOE®-X-100 | N | 0.015 | 2% | 6.38 E+06 |
| 13 | ANAPOE®-X-114 | N | 0.011 | 2% | 5.01 E+06 |
| 14 | ANAPOE®-X-305 | N | - | 2% | 754019 |
| 15 | ANAPOE®-X-405 | N | 0.16 | 2% | 637113 |
| 16 | Big CHAP | N | 0.25 | 2% | 1.36 E+06 |
| 17 | Big CHAP, deoxy | N | 0.12 | 2% | 1.18 E+06 |
| 18 | CYGLU®-3 | N | 0.86 | 2% | 1.27 E+06 |
| 19 | CYMAL®-4 | N | 0.37 | 2% | 2.91 E+06 |
| 20 | CYMAL®-5 | N | 0.12 | 2% | 2.61 E+06 |
| 21 | CYMAL®-6 | N | 0.028 | 2% | 2.90 E+06 |
| 22 | CYMAL®-7 | N | 0.0099 | 2% | 2.73 E+06 |
| 23 | 2,6-Dimethyl-4-heptyl-β-D-maltopyranoside | N | 1.2 | 2% | 1.69 E+06 |
| 24 | 2-Propyl-1-pentyl maltopyranoside | N | 1.9 | 2% | 4.58 E+06 |
| 25 | MEGA-8 | N | 2.5 | 2% | 2.53 E+06 |
| 26 | n-Octyl-β-D-glucopyranoside | N | 0.53 | 2% | 4.01 E+06 |
| 27 | n-Nonyl-β-D-glucopyranoside | N | 0.2 | 2% | 3.59 E+06 |
| 28 | n-Octyl-β-D-maltopyranoside | N | 0.89 | 2% | 2.63 E+06 |
| 29 | n-Nonyl-β-D-maltopyranoside | N | 0.28 | 2% | 2.48 E+06 |
| 30 | n-Decyl-α-D-maltopyranoside | N | - | 2% | 2.28 E+06 |
| 31 | n-Decyl-β-D-maltopyranoside | N | 0.087 | 2% | 2.44 E+06 |
| 32 | n-Undecyl-α-D-maltopyranoside | N | 0.029 | 2% | 2.29 E+06 |
| 33 | n-Undecyl-β-D-maltopyranoside | N | 0.029 | 2% | 2.30 E+06 |
| 34 | n-Dodecyl-α-D-maltopyranoside | N | 0.0076 | 2% | 2.21 E+06 |
| 35 | n-Dodecyl-β-D-maltopyranoside | N | 0.0087 | 2% | 2.72 E+06 |
| 36 | n-Tridecyl-β-D-maltopyranoside | N | 0.0017 | 2% | 2.55 E+06 |
| 37 | n-Heptyl-β-D-thioglucopyranoside | N | 0.85 | 2% | 923303 |
| 38 | n-Octyl-β-D-thiomaltopyranoside | N | 0.4 | 2% | 4.96 E+06 |
| 39 | n-Nonyl-β-D-thiomaltopyranoside | N | 0.15 | 2% | 2.59 E+06 |
| 40 | n-Decyl-β-D-thiomaltopyranoside | N | 0.045 | 2% | 2.27 E+06 |
| 41 | n-Undecyl-β-D-thiomaltopyranoside | N | 0.011 | 2% | 2.63 E+06 |
| 42 | n-Dodecyl-β-D-thiomaltopyranoside | N | 0.0026 | 2% | 2.28 E+06 |
| 43 | Hexaethylene glycol monooctyl ether (C8E6) | N | 0.39 | 2% | 2.27 E+06 |
| 44 | Octaethylene glycol monododecyl ether (C12E8) | N | 0.0048 | 2% | 1.99 E+06 |
| 45 | Pentaethylene glycol monodecyl ether (C10E5) | N | 0.031 | 2% | 2.48 E+06 |
| 46 | Tetraethylene glycol monooctyl ether (C8E4) | N | 0.25 | 2% | 3.59 E+06 |
| 47 | Sucrose monododecanoate | N | 0.016 | 2% | 3.85 E+06 |
| 48 | Dimethyldecylphosphine oxide | N | 0.1 | 2% | 2.31 E+06 |
| 49 | HEGA-10 | N | 0.26 | 2% | 577341 |
| 50 | NP-40 | N | 0.05-0.3 | 2% | 553933 |
| 51 | Digitonin | N | - | 2% | 1.58 E+06 |
| 52 | Deoxycholic acid, sodium salt | A | 0.24 | 2% | 1.37 E+06 |
| 53 | Sodium cholate | A | 0.41 | 2% | 5.24 E+06 |
| 54 | FOS-MEA®-8 | A | 0.59 | 2% | 3.96 E+06 |
| 55 | FOS-MEA®-10 | A | 0.15 | 2% | 3.85 E+06 |
| 56 | Sodium dodecanoyl sarcosine | A | 0.42 | 2% | 1.34 E+06 |
| 57 | Decyltrimethylammonium chloride | C | 0.07 | 2% | 362654 |
| 58 | Dodecyltrimethylammonium chloride | C | 0.0012 | 2% | 437111 |
| 59 | Hexadecyltrimethylammonium chloride | C | 0.000102 | 2% | 954116 |
| 60 | Tetradecyltrimethylammonium chloride | C | 0.0009 | 2% | 1.25 E+06 |
| 61 | ANZERGENT® 3-10 | Z | 1.2 | 2% | 4.14 E+06 |
| 62 | ANZERGENT® 3-12 | Z | 0.094 | 2% | 3.60 E+06 |
| 63 | ANZERGENT® 3-14 | Z | 0.007 | 2% | 2.91 E+06 |
| 64 | CHAPS | Z | 0.49 | 2% | 3.08 E+06 |
| 65 | CHAPSO | Z | 0.5 | 2% | 2.96 E+06 |
| 66 | C-DODECAFOS™ | Z | 0.77 | 2% | 5.32 E+06 |
| 67 | CYCLOFOS™-4 | Z | 0.45 | 2% | 3.24 E+06 |
| 68 | CYCLOFOS™-5 | Z | 0.15 | 2% | 4.39 E+06 |
| 69 | CYCLOFOS™-6 | Z | 0.094 | 2% | 3.92 E+06 |
| 70 | CYCLOFOS™-7 | Z | 0.022 | 2% | 4.98 E+06 |
| 71 | FOS-CHOLINE®-9 | Z | 1.2 | 2% | 2.99 E+06 |
| 72 | FOS-CHOLINE®-10 | Z | 0.35 | 2% | 3.89 E+06 |
| 73 | FOS-CHOLINE®-11 | Z | 0.062 | 2% | 4.25 E+06 |
| 74 | FOS-CHOLINE®-12 | Z | 0.047 | 2% | 5.51 E+06 |
| 75 | FOS-CHOLINE®-13 | Z | 0.027 | 2% | 5.57 E+06 |
| 76 | FOS-CHOLINE®-14 | Z | 0.0046 | 2% | 5.77 E+06 |
| 77 | FOS-CHOLINE®-15 | Z | 0.0027 | 2% | 5.54 E+06 |
| 78 | FOS-CHOLINE®-16 | Z | 0.00053 | 2% | 4.25 E+06 |
| 79 | FOS-CHOLINE®-ISO-9 | Z | 0.99 | 2% | 721324 |
| 80 | FOS-CHOLINE®-ISO-11 | Z | 0.9 | 2% | 1.93 E+06 |
| 81 | FOS-CHOLINE®-ISO-11-6U | Z | 0.87 | 2% | 599655 |
| 82 | FOS-CHOLINE®-UNSAT-11-10 | Z | 0.21 | 2% | 7.65 E+06 |
| 83 | FOSFEN™-9 | Z | 0.014 | 2% | 3.41 E+06 |
| 84 | NOPOL-FOS™ | Z | 1.4 | 2% | 1.85 E+06 |
| 85 | PMAL™-C8 | Z | - | 2% | 3.16 E+06 |
| 86 | PMAL™-C10 | Z | - | 2% | 1.17 E+06 |
| 87 | n-Decyl-N,N-dimethylglycine | Z | 0.46 | 2% | 1.97 E+06 |
| 88 | n-Dodecyl-N,N-dimethylglycine | Z | 0.041 | 2% | 2.43 E+06 |
| 89 | n-Dodecyl-β-iminodipropionic acid,monosodium salt | Z | - | 2% | 2.94 E+06 |
| 90 | n-Tetradecyl-N,N-dimethylamine-N-oxide (TDAO) | Z | 0.0075 | 2% | 3.59 E+06 |
| 91 | n-Dodecyl-N,N-dimethylamine-N-oxide (DDAO) | Z | 0.0023 | 2% | 3.73 E+06 |
| 92 | Digitonin (8%) + Cholate (2%) | M |  |  | 3.12 E+06 |
| 93 | CHAPS (6%) + CHS (1.2%) + DDM (10%) | M |  |  | 3.94 E+06 |
| 94 | CHAPS (10%) + CHS (2%) + DDM (2%) | M |  |  | 3.64 E+06 |
| 95 | CHAPS (10%) + CHS (2%) | M |  |  | 3.45 E+06 |
| 96 | CHAPS (10%) + OG (1%) | M |  |  | 5.15 E+06 |

1. Detergent type: N (non-ionic detergent), A (anionic detergent), C (cationic detergent), Z (zwitter-ionic detergent) and M (detergent mixture);

2. CMC (critical micelle concentration) in H_2_O from www.anatrace.com.

3. Concentration of detergents used during the detergent screen.

4. The intensity of each dot quantified by spot densitometry after Dot blot analysis against rho1D4 tag.
